# Contrast-enhanced ultrasound as a valuable tool to detect minimal inflammation in RA patients in sustained remission

**DOI:** 10.3389/fmed.2024.1459802

**Published:** 2024-12-20

**Authors:** Joaquim Polido-Pereira, Manuel S. António, Nikita Khmelinskii, Marta Arese, Rui Teixeira, Elsa Vieira-Sousa, Maria A. D'Agostino, João E. Fonseca

**Affiliations:** ^1^Rheumatology Department, Unidade Local de Saúde de Santa Maria, Lisbon, Portugal; ^2^GIMM – Gulbenkian Institute for Molecular Medicine, Lisbon, Portugal; ^3^Universitá Cattolica del Sacro Cuore—Foundation Policlinico Universitario Agostino Gemelli IRCCS, Rome, Italy; ^4^Rheumatology Department Catholic University of Sacred Hearth, Foundation Policlinico Universitario Agostino Gemelli IRCCS, Rome, Italy

**Keywords:** rheumatoid arthritis, remission, ultrasonography, microbubbles, synovium

## Abstract

**Objective:**

The study aimed to explore the utility of contrast-enhanced ultrasound (CEUS) as a tool for detecting minimal inflammation in rheumatoid arthritis (RA) patients in sustained remission (SR) and to correlate the findings with Disease Activity Score 28 (DAS28) status scores and various ultrasound (US) scores.

**Patients and methods:**

Thirty RA patients in SR (minimum 6 months), 12 with active disease, and 10 healthy controls were included. Clinical evaluations and US assessments were performed, including grayscale US (GSUS), power Doppler US (PDUS), and Global OMERACT-EULAR Synovitis Score (GLOESS). The CEUS was performed in the two most active joints and was scored semi-quantitatively (SQ) and quantitatively.

**Results:**

Healthy controls and remission RA patients had similar total US scores. Active RA patients had higher US scores than the healthy and remission groups, with statistically significant differences in all the groups compared to the healthy group. However, significant differences were only observed in the GSUS and GLOESS when comparing active RA patients with the remission group. Ninety-five joints were selected for the CEUS, and we detected more microvascularization with the SQ CEUS score than with the PDUS in all groups (18 vs. 58% in the remission group; *p*-value 0.006). The weighted Cohen's kappa for the intra-rater and inter-rater IACUS CEUS score was 0.714 (confidence interval 0.610–0.819, *p*-value < 0.001) and 0.540 (confidence interval: 0.419–0.662, *p*-value < 0.001), respectively. Spearman's correlation between the SQ CEUS and quantitative scores was 0.655.

**Conclusion:**

For the majority of RA patients in SR, conventional US may fail to detect microvascularization potentially related to the subclinical disease. The CEUS may be helpful for this purpose.

## 1 Introduction

Rheumatoid arthritis (RA) is a chronic systemic inflammatory disease that causes joint damage, disability, and increased comorbidities. Although a cure is impossible, remission is an achievable target for the majority of RA patients. Effective monitoring of RA is crucial for optimizing treatment outcomes, preventing disease progression, and improving patient quality of life. Rheumatologists use established instruments to define remission, such as the disease activity score 28 (DAS28), the simplified disease activity index (SDAI), or the clinical disease activity index (CDAI) (1). However, several studies have shown that these tools may miss the detection of minimal inflammation related to a clinically significant risk of relapse or progressive joint structural damage (2).

Multiple treatments, including conventional synthetic (cs), biological (b), and targeted synthetic (ts) disease-modifying anti-rheumatic drugs (DMARDs), are available for RA patients. Due to an earlier diagnosis and the variety of available treatments, a greater number of patients are achieving remission, making treatment tapering an achievable goal for a progressively larger group of patients (3). However, tapering and eventual discontinuation of treatment based only on clinical scores have been shown to increase the risk of relapse (4). The use of serological inflammatory markers is also unreliable, as nearly half of RA patients with normal CRP have shown histological signs of inflammation in the synovial tissue (5). In some patients, this histological inflammation could be due to alternatively activated macrophages characterized by an anti-inflammatory profile, which facilitates the resolution of inflammation and tissue repair (6).

Over the last 20 years, ultrasound (US) has been commonly used in RA patients for diagnosis, disease monitoring, and prognosis (2). Although grayscale US (GSUS) can reflect synovial hypertrophy and effusion and related scores diminish after treatment (7, 8), the use of the GSUS score validated by the OMERACT US group (9, 10) shows that healthy subjects may also have some degree of synovial hypertrophy and effusion in some joints (11). The use of Doppler ultrasound (DUS) has demonstrated a better prediction of treatment response and structural damage when compared to GSUS alone or magnetic resonance imaging (MRI), even in patients in remission (12–15). More recently, a Global EULAR-OMERACT Synovitis Score (GLOESS), which combines GSUS and DUS, has been developed. It shows high discriminative capability and sensitivity to change, improving the identification of patients who are responding to treatment and achieving remission (9, 10, 16).

In RA patients with active disease, both GSUS and DUS, but especially the latter, correlate with several histological synovium features, such as the Krenn score (KS), the T-cell and macrophage infiltration, the cell proliferation marker Ki67, and the vessel wall von Willebrand factor expression. In some studies, the correlation between histology and DUS was even higher than with MRI (16–18).

However, the data are scarcer and more conflicting regarding RA patients in remission. Expression of markers identifying macrophages (CD68), B cells (CD20), T cells (CD3), and endothelial cells (CD31) appears lower in DUS-negative patients in remission than in patients with active disease. Still, these differences disappear in DUS-positive patients (19, 20). Of interest, almost one-third of RA patients in clinical remission show synovium inflammation at the histological and transcriptional levels (21).

Contrast-enhanced ultrasound (CEUS) involves the use of microbubble contrast agents intravenously to enhance the scattering reflection from blood in order to increase the sensitivity to detect microvessels (22). Microbubble ultrasound contrast agents have been available for ~20 years and were initially used for opacification of cardiac chambers and, more recently, to study tumor perfusion or vesicoureteric reflux in children through the intravesical route. These agents are safe and easy to use (3, 23).

Although there are no correlation studies between the synovium histology and the CEUS in RA, there is evidence that several parameters of the CEUS are associated with focal angiogenesis (as marked by CD31) in the synovium of psoriatic arthritis patients (24). In animal models of arthritis, the CEUS was better correlated with synovium vascularity when compared with DUS (25, 26).

Based on a better visualization of the microvascular compartment, the CEUS may be superior to DUS in translating a dynamic process such as synovitis in RA, which may be explained by better quantitative evaluation and less motion artifact and more sensitive to detect subclinical inflammation than DUS in RA patients in DAS28 remission (22, 24). Considering this evidence, we hypothesize that the CEUS might be a better tool to assess imaging remission in RA than DUS.

## 2 Objectives

The main objective was to evaluate the utility of the CEUS in detecting minimal inflammation in rheumatoid arthritis (RA) patients in sustained remission (SR). This was done by correlating CEUS parameters measured using both the semi-quantitative IACUS (International Arthritis Contrast Ultrasound Score) and the VueBox^®^ quantification tool, with GSUS, power Doppler ultrasound (PDUS), and GLOESS parameters in RA patients who had been in clinical remission for at least 6 months [DAS28 4 variables (4v), C-reactive protein (CRP) < 2.6]. These results were compared to those of RA patients with active disease (DAS28 ≥ 2.6) and healthy controls.

The secondary endpoint was to compare the total synovitis score (28 joints) using GSUS, PDUS, and GLOESS in RA patients who had been in clinical remission for at least 6 months with RA patients having active disease and healthy controls.

## 3 Methods

Consecutive patients with RA in clinical remission (target *n* = 30) for at least three consecutive visits (DAS28 4v-CRP) over the previous 6 months and with active US synovitis (GSUS or PDUS grade ≥ 1) in at least one joint during a bilateral evaluation of wrists, metacarpophalangeal joints (1–5), interphalangeal joint of the first finger, proximal interphalangeal joints (2–5), elbows, knees, and tibiotalar and metatarsophalangeal joints (2 and 5) were considered eligible for the study. Additionally, 12 age- and sex-matched RA patients with active disease (DAS28 4v-CRP ≥ 2.6), and 10 age- and sex-matched healthy controls were recruited as positive and negative control groups, respectively. The enrollment period was from December 2018 up to April 2023. The complete medical history was recorded, including demographics, diagnosis date, rheumatoid factor (RF), anti-citrullinated peptide antibodies (ACPA) positivity, erosive disease, and drug intake. All clinical evaluations and the US were performed at the Department of Rheumatology outpatient clinic, Unidade Local de Saúde de Santa Maria, Lisbon, Portugal. This study complies with the Declaration of Helsinki, and the locally appointed ethics committee has approved the research protocol (Approval no. 22/17 from Centro Académico de Medicina de Lisboa Ethics committee). Written informed consent was obtained from all the subjects.

### 3.1 Clinical evaluation

All study participants were evaluated at a single time point. Clinical data were registered in the Rheumatic Diseases Portuguese Registry (Reuma.pt) (27). A total of 68 tender and 66 swollen joints were assessed by two experienced rheumatologists, who were blinded to the US results, by applying a binary scale (present or absent). Furthermore, visual analog scales (VAS) for pain and patient and physician global assessments of disease activity were also collected. ESR and CRP were measured. DAS28 4v-CRP, DAS28 4v-ESR, SDAI, and CDAI were calculated.

### 3.2 Ultrasound evaluation

The GSUS, PDUS, GLOESS, and CEUS parameters were collected by one ultrasonographer (>10 years of experience) on the same day of the clinical evaluation, unaware of clinical findings and disease activity. The ultrasound examinations were conducted using a GE Logiq E9 machine equipped with a 6–15-MHz matrix linear probe. The PDUS was used to assess the vascularization of the synovial tissue. The Doppler parameters were adjusted at the maximum sensitivity for slow flow (pulse repetition frequency of 0.4 kHz, lowest wall filter of 45 Hz, and 7.5–10 MHz Doppler frequency) and Doppler gain adjusted just below the noise level. All examinations were performed using standardized dorsal and dorsolateral scans. The images were stored in DICOM format. The GSUS, PDUS, and GLOESS grading was performed on static images by a blinded senior ultrasonographer based on the EULAR-OMERACT consensus scores (9, 10, 28).

A total score for the GSUS, PDUS, and GLOESS was calculated by summing the individual scores of 28 joints (14 pairs): the wrist, metacarpophalangeal (1–5), interphalangeal joint of the first finger of the hands, proximal interphalangeal hand joints (2–5), knees, and metatarsophalangeal joints (2 and 5). The scores of the 28 studied joints ranged between 0 and 84.

**Figure 1:**
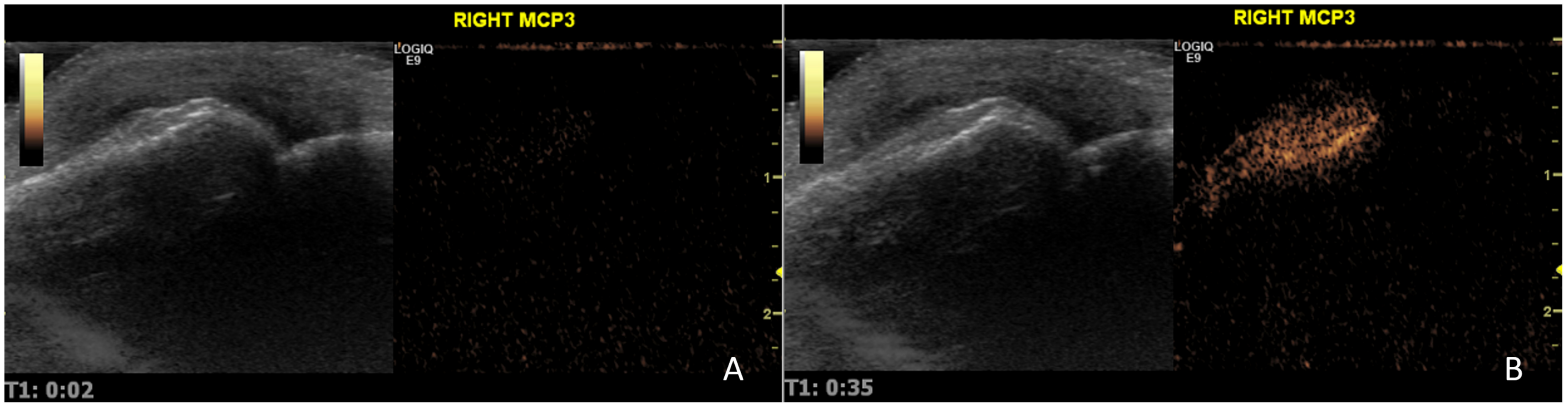
CEUS evaluation of a metacarpophalangeal joint at 2 s **(A)** and near peak, 35 s **(B)**. This joint was classified as having an IACUS score grade 2.

**Figure 2:**
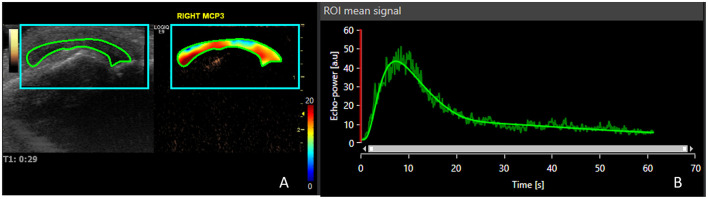
Contrast-enhanced ultrasound quantification using VueBox^®^ software of the same joint in Figure 1. **(A)** The region of interest is depicted, and the heat map shows the contrast enhancement through the 90 s clip after contrast arrival; **(B)** time–intensity curve allowing the determination of signal intensity maximum and minimum.

Up to 2 of 32 joints (16 pairs) were selected for the CEUS study: wrist, metacarpophalangeal joints (1–5), interphalangeal joint of the first finger of the hands, proximal interphalangeal hand joints (2–5), elbow, knee, tibiotalar, and metatarsophalangeal joints (2 and 5). The selection was based on the joints with the highest PDUS score. If PDUS was 0 in all joints, the choice was based on the highest GSUS score. The CEUS was performed after intravenous injection of contrast medium (SonoVue, Bracco International). The mechanical index of the equipment was low (0.1). SonoVue was injected in the dose of 2.4 mL per joint studied, followed by 10 cc of saline as an intravenous bolus in the antecubital vein. The examination was carried out with a frequency of 15 frames per second. A 90 s video clip was started at the beginning of the bolus and was recorded and stored in DICOM format for subsequent review by a senior ultrasonographer blinded for clinical and other US data. A semi-quantitative score was applied according to a scale suggested by the IACUS group (0—no contrast enhancement; 1—contrast enhancement similar to the surrounding small parts; 2—contrast enhancement higher than the surrounding small parts) as shown in Figure 1 (29). The quantitative analysis was performed by a different ultrasonographer who was unaware of the clinical and the other US scores using VueBox^®^ software. A region of interest of synovial hypertrophy was determined for each evaluated joint, as depicted in Figure 2 in the green area (30). A time–intensity curve (TIC) was produced and started at the contrast arrival automatically calculated by VueBox^®^ software but with human validation. This allowed the determination of the signal intensity maximum (SiMax) and minimum (SiMin) and the calculation of the signal intensity ratio (SiR = SiMax/SiMin), as previously described, although with different software (31).

### 3.3 Sample size calculation and statistical analysis

The sample size calculation was based on the results of one study that compared the CEUS positivity between active and inactive RA patients (100 vs. 3.8%) with 80% power. Assuming that remission patients would be 70%, the estimated sample size (after continuity correction) would be 10 (32–34). In a previous study, the difference in the CEUS positivity between active and inactive joints was 99 vs. 49%. Under the same conditions, this would result in an estimated sample size of 33 (33–35). Missing data were handled using complete-case analysis. The data analysis was performed using IBM SPSS Statistics v28. The data were presented as mean ± standard deviation (SD) for continuous variables. Means from unpaired samples were compared, using independent-samples Student's *t*-test or the Mann–Whitney test according to the type of distribution. Levene's test was applied to verify variance homogeneity in normal distributions. For the comparison of multiple groups, ANOVA or Kruskal–Wallis was used. Qualitative variables were analyzed using chi-square and exact Fisher's test or Cramer's *V*-test, as appropriate. Pearson's or Spearman's correlation coefficients were used to determine the correlation between variables as appropriate for the distribution type. For inter and intra-rater evaluation, the weighted Cohen's kappa was used. *P*-values < 0.05 were considered significant after applying Bonferroni correction when suitable.

## 4 Results

### 4.1 Clinical evaluation

Fifty-eight RA patients followed in a remission cohort were screened to be included in the present study. Seventeen patients did not meet the inclusion criteria because they had not achieved remission at one of the visits in the previous 6 months, and 11 refused to participate. The remaining 30 RA patients recruited had a DAS28 4v-CRP < 2.6. The average remission duration was 23.3 ± 18.5 months (range: 6–76.1 months). Additionally, 12 patients with active RA (including low, moderate, and high disease activity) and 10 HCs were also recruited. At the time of evaluation, the distribution of clinical parameters of remission and activity among the 42 RA patients allowed to distribute them as follows: 30 in the remission group and 12 in the active group [four were in low disease activity, three in moderate disease activity, and four in high disease activity (DAS28 4v-CRP)]. Patients' characteristics are shown in Table 1. The disease duration of the active group was statistically significantly lower when compared to the remission group. The active disease group was also more frequently treated with tocilizumab and NSAIDs and was more frequently seronegative for rheumatoid factor. All other patient and disease characteristics and therapy features were similar across all RA groups.

**Table 1:** Participant characteristics.

	**Healthy (*n* = 10)**	**Remission RA (*n* = 30)**	**Active RA (*n* = 12)**	***p*-value**
		**DAS28 4v-CRP < 2.6**	**DAS28 4v-CRP ≥2.6**	
Age, years (mean ± SD)	58.6 ± 11.1	59.0 ± 10.8	64.4 ± 10.9	0.313^*^
Gender (F %)	70	73.3	75	0.965^+^
Disease duration, years (mean ± SD)		7.1 ± 5.9	3.0 ± 4.0	**0.010** ^**x**^
DAS28 4v-CRP (mean ± SD)		1.7 ± 0.3	4.6 ± 1.5	**< 0.001** ^**x**^
DAS28 4v-ESR (mean ± SD)		2.3 ± 0.6	5.2 ± 1.4	**< 0.001** ^**x**^
SDAI (mean ± SD)		2.9 ± 1.7	25.5 ± 15.7	**< 0.001** ^**x**^
CDAI (mean ± SD)		2.6 ± 1.8	23.1 ± 13.5	**< 0.001** ^**x**^
RF (%)		86.7	58.3	**0.043** ^**+**^
ACPA (%)		76.7	66.7	0.505^+^
Erosive (%)		50	33.3	0.327^+^
**Treatment (%); dosage (mean, mg)**				
Methotrexate ORAL		43.3; 16.9	16.7; 11.3	0.103^+^
Methotrexate SC		43.3; 20.8	33.3; 21.3	0.551^+^
Leflunomide		3.3; 20		0.522^+^
Hydroxychloroquine		13.3; 350	16.7; 400	0.780^+^
Sulfasalazine		3.3; 2000		0.522^+^
Tocilizumab		6.7; 162	33.3; 162	**0.026** ^**+**^
Etanercept		3.3; 50		0.522^+^
Prednisolone		43.3; 4.3	33.3; 4	0.551^+^
Deflazacort		3.3; 6		0.522^+^
NSAID^*^		3.3	50	**< 0.001** ^+^

RA, rheumatoid arthritis; DAS28, disease activity score, 28 joints; 4v, four variables; CRP, C reactive protein; F, female; SD, standard deviation; RF, rheumatoid factor; ACPA, anti-citrullinated peptide antibodies; mg, milligrams; SC, subcutaneous; NSAID, non-steroidal anti-inflammatory drugs.

^*^ANOVA.

^+^Cramer's V.

^x^Mann–Whitney; statistically significant results have the *p*-value bolded.

### 4.2 GSUS, PDUS, and GLOESS scores

Thirty-nine RA patients and 10 healthy controls had 28 joints scanned by an experienced ultrasonographer to calculate the total ultrasound scores. In three RA patients with active disease, the total score was not calculated due to technical problems.

Table 2 shows the synovitis scores of the 1,372 joints scanned for the total EULAR-OMERACT ultrasound scores.

**Table 2:** Synovitis score of the joints scanned per study group.

	**Healthy (*****n*** = **10)**				**Remission RA (*****n*** = **30)**				**Active RA (*****n*** = **9)**			
**Grade**	**0**	**1**	**2**	**3**	**0**	**1**	**2**	**3**	**0**	**1**	**2**	**3**
GSUS	196	75	9	0	599	195	41	5	158	59	26	9
PDUS	279	1	0	0	823	9	6	1	236	14	3	0
GLOESS	196	75	9	0	599	195	40	6	158	59	26	9

GSUS, grayscale; PDUS, power Doppler; GLOESS, combined score.

Only 1 out of 280 (0.4%) joints scanned in the healthy controls was PDUS positive, whereas 30% had at least grade 1 synovitis in GSUS. Regarding PDUS in the remission group, only 16 out of 839 (1.9%) scanned joints were positive. In the active group, the number of PDUS-positive joints increased to 17 out of 253 (6.7%) joints.

The total US scores of the healthy controls (Table 3) were statistically significantly different from the active RA group but not from the remission group. The remission group had numerical values closer to the healthy controls than the active group. The GSUS scores overlapped with the GLOESS almost perfectly. The only discrepancy was observed in a metacarpophalangeal joint with GSUS of 2 and PDUS of 3, which led to a GLOESS of 3.

**Table 3:** Total ultrasound scores per group.

**(Avg ±SD)**	**Healthy (*n* = 10)**	**REM (*n* = 30)**	**ACT (*n* = 9)**	**Significance**		
				**H vs. REM**	**H vs. ACT**	**REM vs. ACT**
GSUS	9.3 ± 3.6	9.7 ± 5.2	15.3 ± 7.5	0.405^*^	**0.025** ^*^	**0.031** ^*^
PDUS	0.1 ± 0.3	0.7 ± 1.4	2.2 ± 2.5	0.503^+^	**0.018** ^**+**^	0.135^+^
GLOESS	9.3 ± 3.6	9.8 ± 5.2	15.3 ± 7.5	0.398^*^	**0.025** ^*^	**0.032** ^*^

Avg, average; SD, standard deviation; REM, RA patients in remission; ACT, RA patients in low/moderate/high disease activity; GSUS, grayscale ultrasound score; PDUS, power Doppler ultrasound score; GLOESS, combined OMERACT-EULAR ultrasound score.

^*^Student's *t*-test after Levene's test confirming variance homogeneity with unilateral *p*-value.

^+^Kruskal–Wallis test applied after confirming non-normal distribution, significance after Bonferroni correction. Statistically significant results have the *p*-value bolded.

If we look only at the percentage of patients having at least one joint PDUS-positive, as shown in Table 4, we can observe the clear difference between all groups, ranging from 10% in healthy controls to 75% in the active RA group.

**Table 4:** Subjects with at least one Doppler-positive joint per study group.

***n* (%)**	**Healthy**	**Remission**	**Active**
Doppler negative	9 (90%)	20 (67%)	3 (25%)
Doppler positive	1 (10%)	10 (33%)	9 (75%)
Total	10	30	12

Cramer's V 0.005.

### 4.3 CEUS evaluation

Ninety-five joints were studied using the CEUS, 75 from RA patients and 20 from healthy controls. Healthy control joints were paired considering the three most frequent joints examined: wrist, metacarpophalangeal, and knee. Only 12 joints (16%) surveyed in RA patients were not paired with healthy controls (data not shown).

The PDUS detected vascularization in 5% of the joints of healthy controls submitted to the CEUS, compared to 18% in the RA patients in remission and 45% with active disease.

As shown in Table 5, the CEUS allowed vascularization detection in more joints when compared to PDUS, using the IACUS semi-quantitative score: 25% in healthy controls' joints, 58% in remission, and 80% in active disease had at least grade 1 IACUS score. No CEUS-negative joint was PDUS-positive (data not shown). The weighted Cohen's kappa for the intra-rater and inter-rater IACUS CEUS score was 0.714 (confidence interval 0.610–0.819, *p*-value < 0.001) and 0.540 (confidence interval 0.419–0.662, *p*-value < 0.001), respectively.

**Table 5:** Power Doppler and contrast-enhanced ultrasound IACUS score in the CEUS studied joints.

**PDUS score**	**Healthy, *n* (%)**	**REM, *n* (%)**	**ACT, *n* (%)**	**IACUS score**	**Healthy, *n* (%)**	**REM, *n* (%)**	**ACT, *n* (%)**
0	19 (95%)	45 (82%)	11 (55%)	0	15 (75%)	23 (42%)	4 (20%)
1	1 (5%)	4 (7%)	3 (15%)	1	4 (20%)	18 (33%)	5 (25%)
2	0	5 (9%)	5 (25%)	2	1 (5%)	14 (26%)	11 (55%)
3	0	1 (2%)	1 (5%)				
Total	20	55	20	Total	20	55	20

Cramer's V p-value of 0.089 for PDUS and 0.002 for IACUS (0.006 and 0.002, respectively, if we consider PDUS and IACUS scores either positive or negative only).

PDUS, power Doppler ultrasound; REM, RA patients in remission; ACT, RA patients in low/moderate/high disease activity; IACUS, International Arthritis Contrast Ultrasound score; CEUS, contrast-enhanced ultrasound.

**Figure 3:**
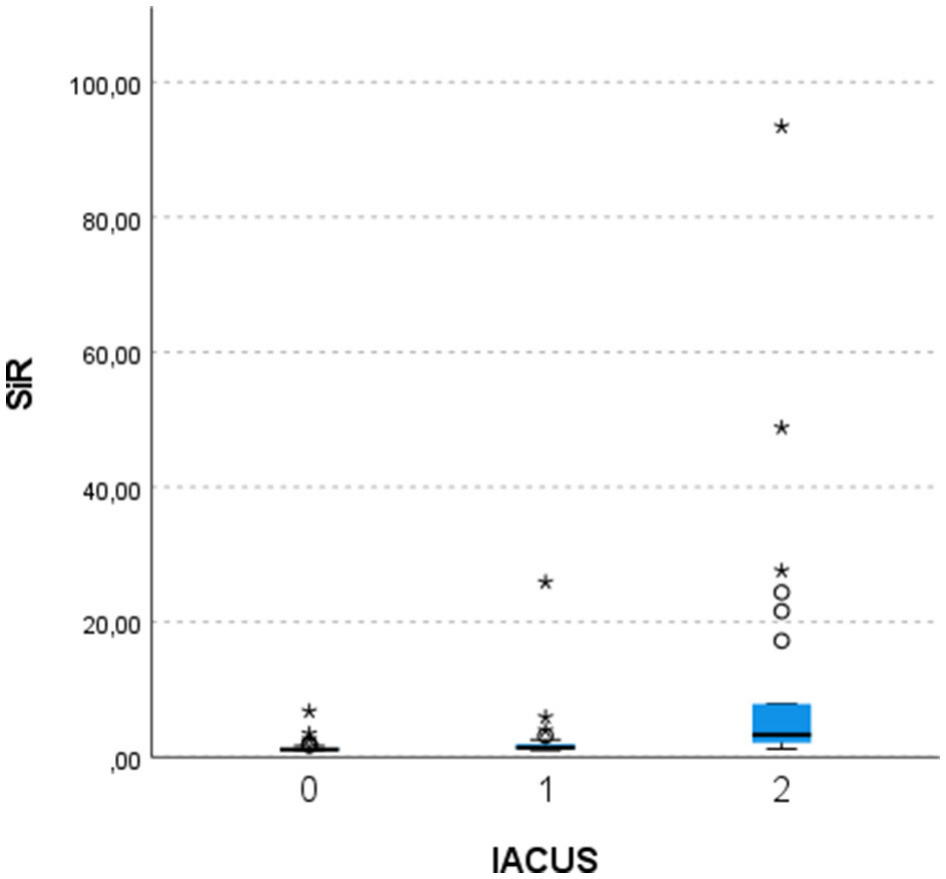
Quantitative score correlation with the semi-quantitative contrast-enhanced ultrasound score. SiR, signal intensity ratio; IACUS, International Arthritis Contrast Ultrasound score; Kruskal–Wallis test, *p*-values after Bonferroni correction: 0 vs. 1–0.004; 0 vs. 2— <0.001; 1 vs. 2–0.013. *outlier values.

Finally, the semi-quantitative IACUS score showed a correlation with the quantitative score and the SiR, and the results are shown in Figure 3. The mean SiRs were 1.4, 2.7, and 11.6 for the IACUS grades 0, 1, and 2, respectively. The median SiRs (interquartile 25–75) were 1.1 (1–1.3), 1.4 (1.2–1.9), and 3.3 (2–10.2), respectively. The correlation coefficient between the IACUS score and SiR obtained using Spearman's Rho was 0.655 (moderate to strong, *p*-value <0.001). The correlations with GSUS, PDUS, and GLOESS with SiRs were as follows: 0.286 (weak, *p*-value: 0.005), 0.509 (moderate, *p*-value: <0.001), and 0.289 (weak, *p*-value: 0.003), respectively.

## 5 Discussion

This study demonstrated that the CEUS IACUS score is a potentially more sensitive method of detecting microvascularization related to subclinical inflammatory activity in RA patients in persistent remission when compared to the established EULAR-OMERACT scores used in clinical practice, namely, GSUS, PDUS, and GLOESS (31). One of the issues we may find by increasing the detection of microvessels in the synovium is finding more normal vessels unrelated to inflammation. To overcome this caveat, we decided to include not only RA patients in remission and active disease but also age- and sex-matched healthy controls. This study is unique as it combined a broad spectrum of synovial phenotypes, ranging from healthy to overt active synovitis. In addition, it is one of the largest studies based on second-generation ultrasound contrast agents performed in RA patients (29).

As shown in the literature, both the CEUS and PDUS depend on RA disease activity. In a previous study, the percentage of the PDUS- and CEUS-positive joints in RA patients in remission were 0 and 3.8%, respectively (32). This contrasts with the results of our study, in which we found PDUS and CEUS positivity of 18 and 58%, respectively, and the results of a more recent study showing 83% CEUS positivity (36). In studies evaluating active joints of RA patients with active disease, the detection of microvascularization increased from 7.4 to 75% with the PDUS to 77.8–100% with the CEUS (the majority of studies reporting 100%). Larger and deeper joints tend to show lower levels of positivity. Quantification methods or differences in equipment quality may explain some of the disparities observed. Nevertheless, the PDUS and CEUS positivity values in the joints studied in the active RA patients from our study (45 and 80%) were similar to what was reported in the literature (29, 31, 32, 37–39).

There is very little information regarding healthy controls in literature when using the same contrast agent. However, one study on coxitis found 0% positivity for both PDUS and CEUS in five healthy controls (39). In contrast, an earlier study showed that the detection of microvascularization in the wrist increased from 73% using color Doppler ultrasound (CDUS) to 91% with the CEUS (using SonoVue), while in the MCP joints, from 18 to 51% (22). In the healthy controls from our study, microvascularization detection increased from 5% with the PDUS to 25% with the CEUS. Different methods, equipment, and studied joints may explain these numerical discrepancies.

Spearman's correlation between the CEUS VueBox quantification of single joints (using the SiR) and the other US scores were 0.655 (moderate to strong), 0.509 (moderate), 0.289 (weak), and 0.286 (weak), for the IACUS CEUS, PDUS, GLOESS, and GSUS, respectively. This aspect strengthens the internal validity of our results. Klauser et al. found a SiR Spearman correlation of 0.37 and 0.4 for the PDUS and IACUS CEUS scores, respectively, lower than in our study. Using older, less optimized quantification software and equipment differences might explain these results (11, 31, 37, 40, 41).

Regarding the secondary endpoint, the total score applied with 28 joints (14 paired) could not differentiate healthy controls from RA patients in remission when looking at the GSUS, PDUS, and GLOESS. A possible explanation for these results was that the findings of synovitis in our healthy controls might be due to other causes of joint inflammation, such as osteoarthritis since patients were matched for age and sex. However, our findings align with a large study on healthy subjects that found 31% synovial hypertrophy (GSUS) in all the evaluated joints (30% detected in our study). In the same study, the PDUS findings were similarly uncommon (11).

The prevalence of PDUS-positive remission RA patients in this study was low (33%) compared to a systematic literature review that found a percentage of 44% (out of 1,369 subjects) (14). The main reason for these findings is related to the fact that the patients in our cohort were in sustained remission for a minimum of 6 months (almost 24 months on average), contrary to the patients of the studies included in the systematic review who had to be in remission only in the specific time point of the US evaluation (14).

Some limitations should be acknowledged. The first one is the definition of remission. We decided to include DAS28 4v-CRP persistent remission, which is a less stringent criterion. This choice was made due to the difficulty in clinical practice to recruit patients in sustained remission using other more strict criteria. Another limitation is the cross-sectional design, which prevents the inference of prognostic information such as treatment response, risk of flare, or structural damage—possible only through longitudinal studies. Additionally, the CEUS can only be used in two joints per patient for ethical reasons, as determined by the SonoVue Summary of Product Characteristics (40). Other US diagnostic modalities that do not need intravenous drug administration, such as superb microvascular imaging (SMI; Toshiba Medical Systems, Tokyo, Japan), are more feasible and allow the study of more joints per patient (41). This tool has proven to be more sensitive than the PDUS and equally sensitive as the CEUS in diagnosing synovitis in RA patients. However, it also lacks evidence of normality in healthy subjects (36, 42). Finally, other comparators, such as histology, could enrich the evidence presented, as it is known that some RA patients in clinical remission show synovium inflammation (21), and some macrophage transcriptional signatures are associated with drug-free remission (6).

For most RA patients in persistent remission, the musculoskeletal ultrasound used in clinical practice may fail to detect microvascularization eventually related to subclinical disease, and the CEUS may be a useful tool for this purpose, although normality cutoffs are not yet clear.

## Data Availability

The raw data supporting the conclusions of this article will be made available by the authors, without undue reservation.
